# Thermal Insulation Based on NBR-Elastomerized Phenolic Resin Reinforced with Carbon Fibers: Mechanical and Ablation Properties

**DOI:** 10.3390/ma18102250

**Published:** 2025-05-13

**Authors:** Jelena Gržetić, Saša Brzić, Slavko Mijatov, Saša Živković, Veselin Živanović, Jela Galović, Tihomir Kovačević

**Affiliations:** 1 Department for Materials and Protection, Military Technical Institute, Ratka Resanovića 1, 11030 Belgrade, Serbia; sasabrzic@gmail.com (S.B.); slavko.mijatov@yahoo.com (S.M.); tkovacevic@tmf.bg.ac.rs (T.K.); 2 Department for Rocket Propulsion, Military Technical Institute, Ratka Resanovića 1, 11030 Belgrade, Serbia; sasavite@yahoo.com (S.Ž.); veselin.zivanovic.best@gmail.com (V.Ž.); 3 Department for Organic-Technical Materials, Military Technical Institute, Ratka Resanovića 1, 11030 Belgrade, Serbia; polimery04@gmail.com

**Keywords:** temperature protection materials, polymer ablatives, NBR blend, thermal behavior

## Abstract

In this paper, thermal and mechanical properties of ablative thermal protective material (TPM) as inhibitors for a free-standing propellant grain based on phenolic resin (PR) and acrylonitrile butadiene rubber (NBR) were investigated. NBR elastomerized PR composite, reinforced with chopped carbon fibers (CFs) (PR/NBR/CF), was prepared by homogenization of 90 parts by weight (PBW) PR in 100 PBW NBR (28 wt.% of acrylonitrile content). PR/NBR/CF composite was blended in two-roller open and closed mixers and in a twin-screw extruder. Carbon black, aluminum(III)-oxide, and fumed silica were added as promoters of thermal and mechanical properties of PR/NBR/CF. The structural analysis was studied using Fourier transform infrared spectroscopy (FT-IR). Thermal properties of the prepared PR/NBR/CF composite inhibitor were studied by ablation and firing tests, while a morphological analysis of the char layer formed after the ablation test was conducted via scanning electron microscopy (SEM). A low erosion rate of 2.00 × 10^−4^ m·s^−1^ and high tensile strength and elongation at break of 6.7 MPa and 419.92%, respectively, indicate that the developed materials can be applied as a thermal insulation/inhibitor of free-standing rocket propellant grains. Bond strength between PR/NBR/CF composite and aluminized composite rocket propellant (ACRP), determined via a standard peel test, showed higher adhesion forces between the PR/NBR/CF composite and the ACRP compared to the cohesion between the ACRP molecular chains.

## 1. Introduction

Development of high-temperature protection materials (TPMs) plays an important role in designing construction elements for rocket motors [1,2]. Depending on the operational conditions (hyperthermal), there are two types of TPMs, non-ablative and ablative, which act as two distinctive thermal protection mechanisms [2,3]. Polymer ablatives are of great importance for solid rocket motor development since these materials have been used for thermal protection of rocket cases, nozzles, etc. They prevent the rocket chamber from being exposed to combustion gases at extremely high temperatures (2400–3700 °C in a very short time) and ensure the integrity of the motor without failure [4,5]. Polymeric materials have been widely used as ablative TPMs due to numerous advantages, such as superior mechanical and thermal properties, low density, and high resistance to thermal variations (low to high temperatures) [2,6].

Elastomeric TPMs should work as flexible heat shielding materials with high elongation at break. The ablation resistance of rubber-based elastomeric TPM composites is reduced compared to the brittle phenolic resin (PR)-based composites [7]. The lower ablation resistance of rubber-based TPMs occurs due to the stresses between the pyrolysis zone and the generated char layer caused by the pyrolysis gases. Pyrolysis gases easily remove the char layer by the action of high-speed incoming shear heat flux [7]. Therefore, the combination of elastomers and thermosets, such as nitrile–butadiene rubber (NBR) and PR, in the form of polymer blends or composites, represents a promising formulation in the design of elastomeric-based TPMs [8,9]. In general, NBR is widely used in elastomeric TPMs because it has good compatibility with resins and fillers, which ensure high char yield (fibers and metal oxides). In addition, NBR provides flexible deformation during propellant combustion [10]. PR is a rigid thermosetting resin that provides high hardness, tensile strength, and carbonization yield. The improvement in mechanical properties of NBR vulcanizates occurs due to the formation of a 3D-network within the material, especially if in situ polymerization of PR in NBR is performed [11]. Iqbal et al. [12] developed NBR-based composites with various amounts of phenolic resin (0, 20, 25, and 30 wt.%) for high-temperature application. They found that the ablation rates, char yield percentage, and insulation indexes decreased with increasing PR content in NBR. Nawaz et al. [13] confirmed that PR remarkably improved the ablation resistance and thermal properties of NBR/PR composite. The linear and mass ablation rates were reduced to 21.3% and 26.1% respectively, with a PR content of 50 phr.

In recent decades, the most commonly used fibers in the design of TPMs have been asbestos fibers, but they have been banned due to negative health effects (carcinogenicity) [14,15]. Glass, aramid, polybenzimidazole, and carbon fibers are the most promising replacement for asbestos fibers [5]. Carbon fibers (CF) are extremely resistant to high temperatures and their melting temperature reaches 4000 °C [16]. CFs are good thermal conductors and reduce mass loss in TPM formulations. Chopped CF in the form of 6 mm long thread, combined with Kevlar pulp and ammonium polyphosphate as a fire retardant, was used as reinforcement for preparing rocket motor insulation based on ethylene propylene diene monomer (EPDM) [4]. It has been found that the formulation containing a mixture of 25 phr CF and 25 phr Kevlar pulp gives the best ablation rate of 0.005 mm·s^−1^, as well as a tensile strength of 9.6 MPa. A similar study using EPDM-based thermal insulation containing equal quantities of CF and aramide fibers (1:1 mass ratio) shows a reduction in the ablation rate to 0.0604 mm·s^−1^, which is 31% lower than that of an insulator filled with AF alone [15].

When CF combined with powdered fillers such as metal/metalloid oxides, carbides etc., the overall thermal reinforcement effect is enhanced. When a rubber matrix contains metal/metalloid oxides together with a phenolic resin, the latter two react to form a protective char, which prevents heat penetration to virgin material [2].

The aim of this study was to develop an ablative thermal insulation for a free-standing rocket propellant grain based on NBR reinforced with PR as a char promoter and chopped CF as a carrier for the mechanical strength of the carbonized layer. The PR/NBR/CF composite was manufactured by a blending technique using a two-roller mixer for compounding the ingredients and a steel cylindrical tool for curing the material (vulcanization). The synergistic effects of PR, CF, and reinforcing fillers (fumed silica and aluminum(III)-oxide) on mechanical and ablation resistance were investigated under laboratory conditions and in experimental static tests of a rocket motor. The results presented in this paper can be used for the development of fire-resistant TPMs based on PR/NBR/CF blends reinforced with inorganic powder fillers.

## 2. Materials and Methods

### 2.1. Materials

All reagents were analytical-grade chemicals. NBR rubber, 28%, phenolic resin–iditol (melting temperature 90 °C, free phenol content ˂2 wt.%, density 1.3 g·cm^−3^) was purchased from Uni Global Ltd., Belgrade, Serbia. Iditol is a novolac type of phenolic resin obtained by condensation of an excess of phenol with formaldehyde in the presence of a hydrochloric acid catalyst. Carbon fibers (3 mm) were purchased from R&G Faserverbundwerkstoffe GmbH, Waldenbuch, Germany. Fumed silica powder, average particle size 0.2–0.3 μm (aggregate), and aluminum(III)-oxide (Al_2_O_3_) were purchased from Sigma Aldrich, Darmstadh, Germany. Carbon black (99.5%), Vulkacit H-30 and Vulkacit DM were purchased from Lanxess, Nagda, India. Stearic acid and sulfur were purchased from Sigma Aldrich, Germany.

Aluminized composite rocket propellant (ACRP), based on hydroxyl-terminated poly(butadiene) (HTPB) prepolymer, was used to investigate bonding strength with developed PR/NBR/CF composite and thermal stability of TPM during static testing of a solid rocket motor. The following chemicals were used: HTPB, commercial name R-45M (USA, viscosity at 30 °C: 5500 mPa·s, OH value: 44–51 mg KOH g^−1^, hydroxyl functionality: 2.4, average molecular weight, Mn: 2900 g mol^−1^, glass transition temperature, T^g^: −80 °C), was used as a prepolymer. Dioctyl adipate (DOA) plasticizer and the bonding agent triethylenetetramine (TETA) were purchased from Merck, Darmstadh, Germany. The antioxidant AO 2246 (2,2′-bis(4-methyl-6-tertbutyl) phenol was supplied from Sigma Aldrich, Germany. Alumina with average particle sizes of 15 and 30 μm (X-71 and X-86) was procured from Alcan Toyo, Naperville, IL, USA. Ammonium perchlorate oxidizer (AP–grade IZ-200-TCP) was procured from Eruca Technologies, Bohumin, Czech Republic. The curing agent isophorone diisocyanate (IPDI) was supplied from Acros Organics, Geel, Belgium.

### 2.2. Blending Technology of PR/NBR/CF Composite

**Figure 1:**
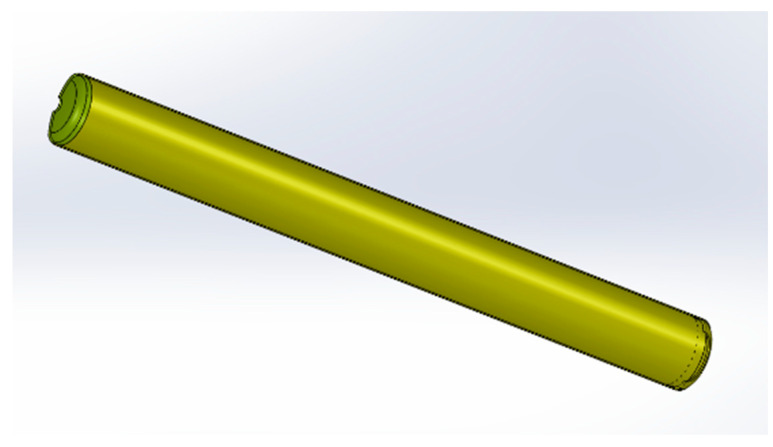
PR/NBR/CF composite thermal insulation for experimental tests of motor rocket firing.

**Scheme 1:**
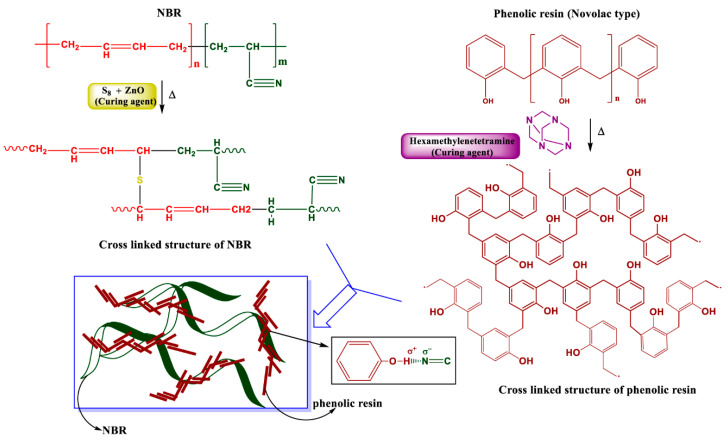
Interpenetrating network of NBR with phenolic resin.

The rubber compounding was carried out in two stages: the first in an open system containing a two-roller mixer and the second in a closed mixer. Afterwards, when the homogenization process was complete, a long-length twin-screw extruder was employed to obtain the material in its final form. The first stage involves the preparation of a basic PR/NBR/CF composite blend without sulphur and accelerators, which were introduced into the formed blend in the second stage. Finally, the mixture was poured into steel cylindrical molds for static tests of rocket motor burning (Figure 1) and for the PR/NBR/CF-ACRP bonding test and cured for 2 h at 160 °C and then 2 h at 180 °C. Table 1 shows the PR/NBR/CF formulation with the addition of fiber and inorganic mineral reinforcements. A schematic representation of the interpenetrating network of NBR with phenolic resin (PR/NBR composite) is shown in Scheme 1. As PR is dispersed in NBR during the mixing process and then polymerized into thermosetting material during vulcanization, the PR formed in situ is in the form of 3D-network structures [11], as shown in Scheme 1. Also, during vulcanization, electrostatic interactions occur between the nitrogen atoms of NBR and the phenolic hydroxyl groups from PR. However, there are still free nitrile and phenolic hydroxyl groups capable of achieving interactions with HTPB polymer chains and IPDI as well.

### 2.3. Homogenization of the Propellant

The propellant was homogenized using a semi-industrial 121/2 PUM Baker-Perkins planetary mixer (Peterborough, UK) at 60 °C. The homogenization process started with a premix phase where the HTPB prepolymer, plasticizer, TETA bonding agent, AO22 antioxidant, and metallic fuel (15 wt.% of 50/50 bimodal aluminum mixture) were placed in the mixer and homogenized for 30 min at 60 °C. The next step included adding the AP oxidizer (≈70 wt.%) in three equal portions. The mixture was homogenized for 90 min at 60 °C under vacuum (10–14 mbar). Finally, the IPDI curing agent was added and the resulting slurry was homogenized for an additional 15 min. The liquid propellant was then cast into a cylindrical mold with dimensions of Ø 120 × 130 mm (bond strength test) and of Ø 170 × 1660 mm (rocket motor static test) and cured in an oven for five days at 70 °C.

### 2.4. Characterization of Materials

The Fourier transform infrared spectroscopy (FT-IR) spectrum of the cured PR/NBR/CF composite was recorded in absorbance mode using a Nicolet™ iS™ 10 FT-IR spectrometer (Thermo Fisher SCIENTIFIC, Waltham, MA, USA) with Smart ATR™ FTIR sampling accessories, within a range of 400–4000 cm^–1^, at a resolution of 4 cm^–1^ and in 20 scan mode.

Scanning electron microscopy (SEM) was performed to observe the dispersion and length of chopped carbon fibers, as well as the morphology of the PR/NBR/CF composite after the oxyacetylene ablation test (SEM, JSM-5900LV, Tokyo, Japan).

The tensile properties and bond strength between PR/NBR/CF composite and ACRP were determined by Instron 1122 Universal Testing Instrument (Instron, Nortwood, MA, USA) at 20 °C with a loading rate of 10 mm·min^−1^.

Morphological analysis of the PR/NBR/CF composite after oxyacetylene ablation testing was performed using SMTV Visor Inspection System (Michael Bruch, Herne, Germany).

Thermal characteristics of the TPM were determined by oxyacetylene ablation testing and static rocket motor firing. The thermal properties of the vulcanized PR/NBR/CF composite were studied using modified oxyacetylene ablation testing using a high-temperature oxyacetylene flame (up to 3000 °C) and infrared thermometer (Extech, Boston, MA, USA). During the test, a high heat flux is generated (Nawaz et al., 2018 [13]). The nozzle diameter was 2.0 mm. Three test replicates were performed. Standard-sized specimens (100 × 100 × 6 mm) were used. The flame/sample distance was 10 mm. The following parameters were obtained as test results: insulation index (IT, Equation (1)); erosion rate (E_in_ m·s^−1^, Equation (2)); t_T_(s) is the time required for the back-face temperature to rice to a certain temperature (T = 80 °C, 180 °C and 380 °C); d (m) is the specimen thickness; and b (s) is the burning time.

(1) $$I_{T}=\frac{t_{T}}{d}$$

(2) $$E=\frac{d}{b}$$

**Figure 2:**
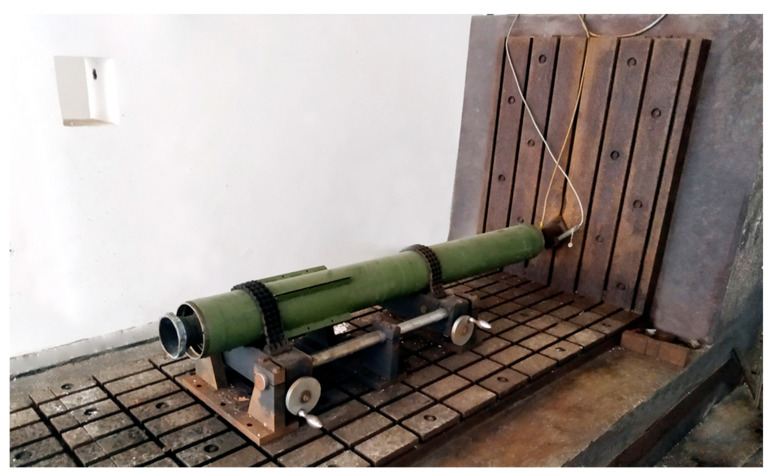
Solid rocket motor stand for static test.

Experimental testing of the rocket motor was performed in the Military Technical Institute specialized laboratory for static tests. The rocket motor was mounted and secured on an adequately designed test stand on a massive concrete platform in a secured special facility intended for hazardous tests (Figure 2). On the test stand, it is possible to test rocket motors up to 200 kN of thrust. The one-axis test stand enables infinitesimal movement of the rocket motor only in the thrust force–axial direction. In that direction, the rocket motor is supported via an adapter for the appropriate force transducer–load cell, which can measure a thrust of up to 50 kN. For pressure measurement, a threaded ring is designed on the front part of the combustion chamber of the rocket motor, in which a pressure transducer is mounted, with a range of up to 500 bar.

The experiment is directed by the chief operator in the control and measurement center, who coordinates the work with pyrotechnicians and security personnel using the communication system. The security of the facility signals readiness for the test, pyrotechnicians connect the ignition wiring of the rocket motor and activate the first safety key, and security activate the second safety key. Then, the operator activates the third safety key and finally triggers the ignition of the rocket motor. After the end of the rocket motor operation process, measured signals from the transducers are recorded using the acquisition system, which consists of measuring transducers, amplifiers, and computers for storing data and processing the results. Recorded data is processed using an originally developed method that is included in the national military standard.

## 3. Results and Discussion

### 3.1. FTIR Analysis

**Figure 3:**
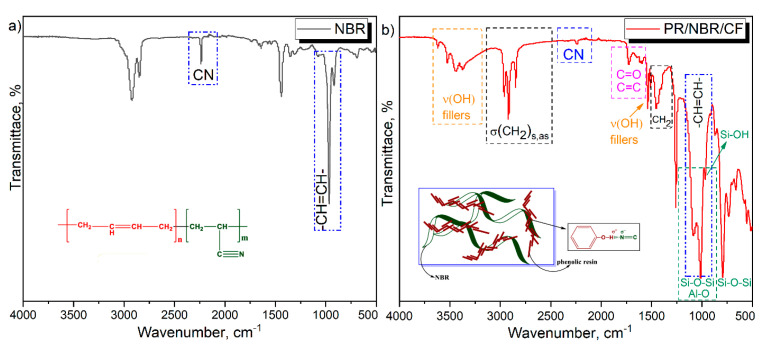
FTIR analysis of (**a**) the NBR and (**b**) the PR/NBR/CF composite.

FTIR analysis is a spectroscopic method suitable for detecting the presence of interactions between the NBR matrix and the PR resin [14]. As shown in Scheme 1, PR forms a 3D-network in NBR during in situ polymerization [11] by making electrostatic interactions as well as hydrogen bonds with nitrogen atoms of the NBR chains. Figure 3 shows the characteristic peak at 968 cm^−1^ corresponding to the vibration of the trans double bond of butadiene (-CH=CH-). The peak at 2240 cm^−1^ corresponds to the nitrile group within NBR, the intensity of which decreases in the obtained composite due to electrostatic interactions with the free phenolic hydroxyl groups, as well as interactions with surface hydroxyl groups of the fillers (SiO_2_ and Al_2_O_3_) (Figure 3b). The remarked peak in the region between 3750–3250 cm^−1^ in the spectrum of the PR/NBR/CF composite is attributed to the stretching vibration of the phenolic hydroxyl group. This peak overlaps with the peaks originating from the vibrations of the surface hydroxyl groups of the fillers (νOH). The peak at 1250 cm^−1^ is attributed to the stretching vibration of phenol (C–OH). Asymmetric and symmetric stretching vibrations of methyl and methylene groups are remarked in the region below 3000 cm^−1^. Numerous peaks between 1800–800 cm^−1^ (Figure 3b) correspond to the enhancement of interactions between the two polymeric domains within the PR/NBR/CF composite. Strong peaks around 1000 cm^−1^ and 750–800 cm^−1^ correspond to Al-O and Si-O-Si vibration.

### 3.2. Mechanical Characterization and Bond Strength

**Figure 4:**
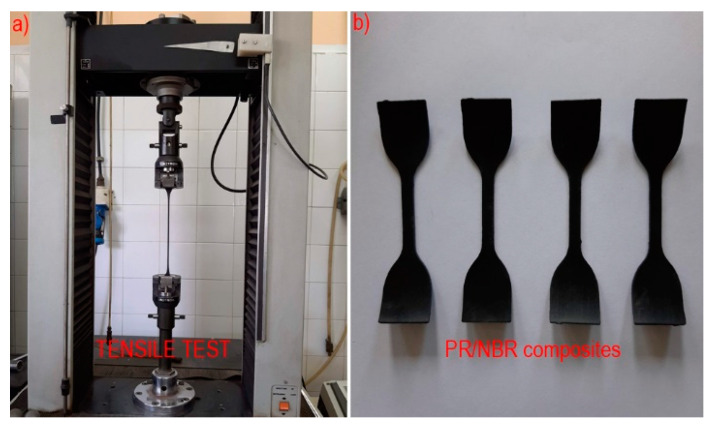
(**a**) Tensile test set-up and (**b**) standard specimens for tensile property determination.

**Figure 5:**
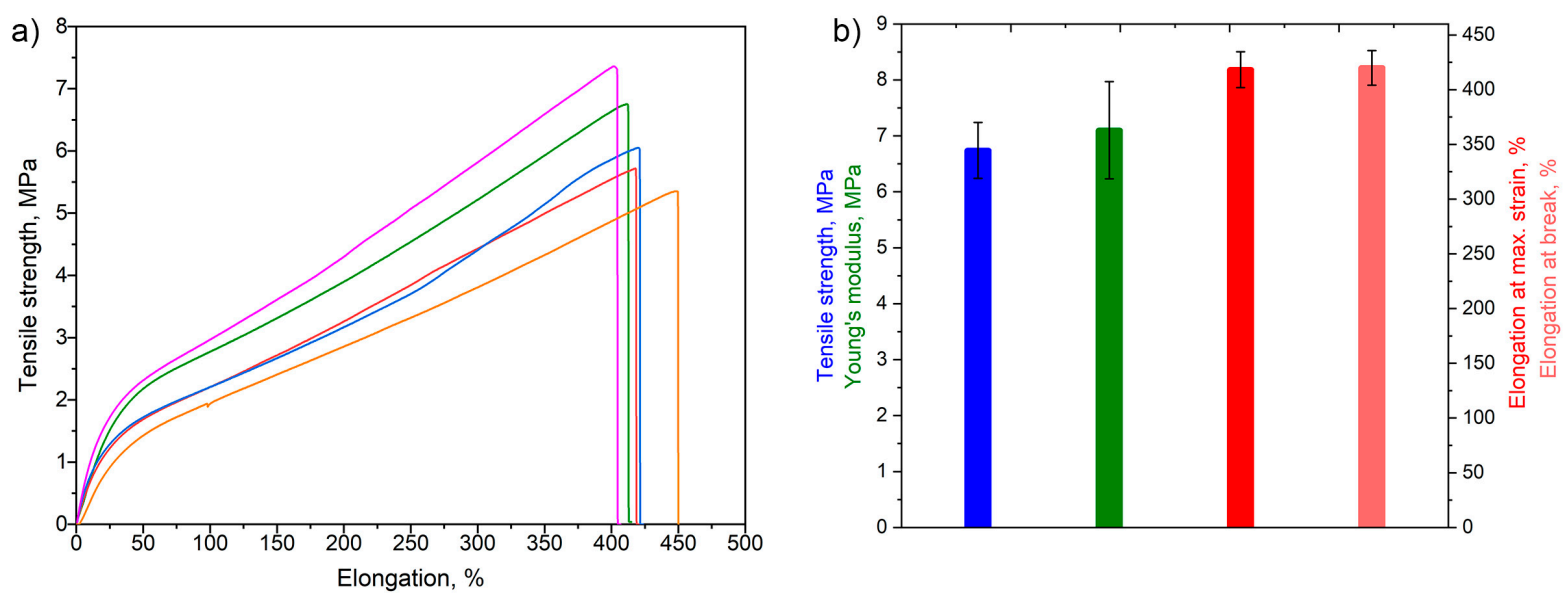
(**a**) Tensile strength–elongation curves and (**b**) average values of mechanical properties for PR/NBR/CF composite.

Tensile tests were performed to determine whether the developed TPMs met the required criteria for application in rocket motors (Figure 4). The PR/NBR/CF formulation was designed based on the fact that in situ polymerized PR is very effective for strengthening NBR vulcanizates [11]. Moreover, the role of CFs was to increase the strength of PR/NBR/CF composite, as well as the strength of the char layer after thermal degradation during the exploitation of the rocket motor. In addition, the incorporation of hard inorganic fillers into NBR elastomer increases the mechanical and thermal integrity of the corresponding composite. However, the optimal level of filler addition provides better strength as well as acceptable elongation at break [11]. The tensile strength–elongation curves of PR/NBR/CF composites are presented in Figure 5. A significant elastomerizing effect was achieved by NBR blending with PR. The analyzed composite shows high values of elongation at maximum strain and break (ε_max_ = 418.3 ± 16.3% and ε_br_ = 419.9 ± 15.9%, respectively). The reached value of tensile strength is lower (σ = 6.7 MPa) compared to similar systems from the available literature [11,14,15]—Table 2. This phenomenon is a consequence of the complex composition of the PR/NBR/CF-based TPM, where the amount of solids (fillers and fibers) is high, above 41 wt.%. This structural complexity is evident in the non-uniformity of the tensile strength values (Figure 5a), which range from 5.3 MPa to 7.4 MPa. The introduction of more than 40 wt.% of thermally stable solids deteriorates the tensile strength due to dissimilarity between powder reinforcements and polymer matrix (inorganic and organic materials). In addition, metallic oxides have a polar surface in contrast to organic resin, making this system incompatible. The high amount of solids in combination with ~26.0 wt.% of phenolic resin makes the NBR-based material rigid and brittle, which is manifested through close values of elongation at maximum strain and elongation at break, i.e., the absence of plastic deformation (Figure 5b). Namely, the phenolic resin creates a structural network upon in situ curing, enriched with numerous bulky and rigid aromatic rings, which, in addition to its quantity, stiffen the corresponding composite, reflected in reduced tensile strength and deformability. However, the reduction in tensile strength is not a disadvantage of this material since it is used for the propellant grain inhibitor, which must not be too strong in order to absorb the stress caused by the rocket rotation during its flight (such a material has to possess good flexibility).

Contrary to this study, Ye et al. [11] showed that both tensile strength and maximum elongation increase almost linearly with increasing PR content up to 15 PBW (σ = 21.0 MPa and ε_max_ = 525.0%). A further increase in PR content causes a slight decrease in both parameters. The authors explained such a phenomenon by the good mechanical bonds between the PR phase and the NBR matrix at lower PR content. Nevertheless, this research does not include an observation of the effect of PR content on the improvement of char yield upon thermal exposure, which is the aim of this research. The NBR-Ph composite, developed by Kumar et al. [14], contains 40 wt.% resole PR resin and 4 layers of carbon fabric in the form of pre-preg, and achieved 17.7 MPa and 668.4% for σ and ε_br_, respectively. Woven carbon fibers improve the mechanical properties and integrity of the material in which it is embedded [17]. Resole PR resin in liquid form is suitable for the preparation of layered pre-pregs but smaller amounts of PR can be impregnated onto the carbon fabric compared to chopped CF used in the PR/NBR/CF composite. The PR/NBR/CF composite is also suitable for loading higher amounts of solid novolac PR resin and CF, and even showing lower values of σ and ε_max_, they are still within the limits accepted for rocket applications, especially if ablation resistance is considered (Section 3.3).

#### Bond Strength Between PR/NBR/CF Composite and ACRP

**Figure 6:**
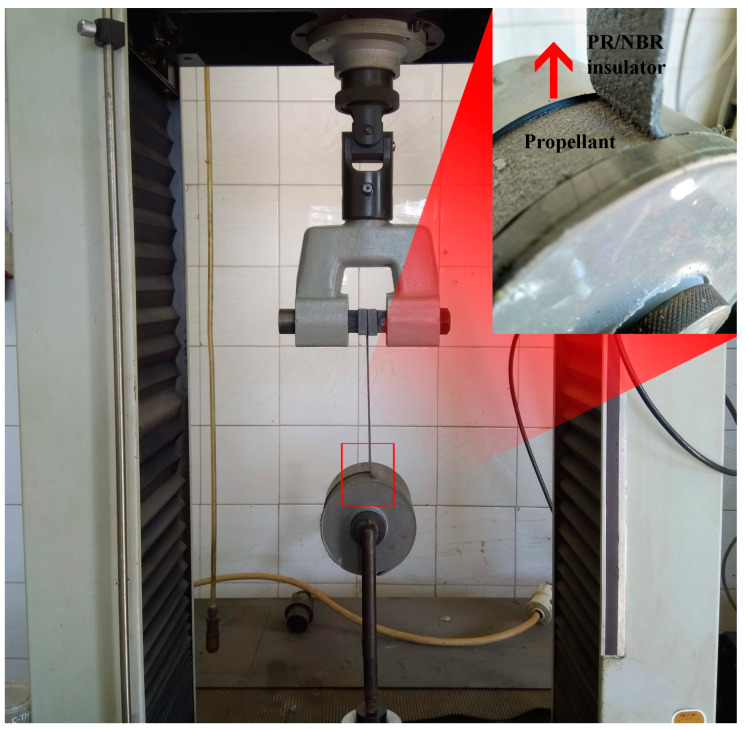
Bond strength test design.

The high bond strength between TPM and propellant is of great importance in the design of rocket propellant grains [17,18]. De-bonding between TPM and propellant causes an uncontrolled increase in the burning area and, consequently, motor failure [17]. PR/NBR/CF-ACRP bond strength tests were performed on a strip cut from a cylindrical casing (Figure 6). The thickness of the PR/NBR/CF composite was 3 mm while the width of the strip was 2.5 cm. The strain rate was 10 mm·min^−1^. The ACRP split during the test, leaving a mark on the PR/NBR/CF strip (insert in Figure 6). This indicates that the bond strength between PR/NBR/CF and ACRP (adhesion forces) is higher than the strength of the ACRP itself (cohesion forces within the propellant structures) [17,18]. This phenomenon is associated with intermolecular interactions (electrostatic and hydrogen bonds) between the free phenolic hydroxyl groups of the PR or free electrons from the nitrile group and untied hydroxyl groups from the polymer matrix of the propellant. The results are given in Table 3.

### 3.3. Ablation Resistance

**Figure 7:**
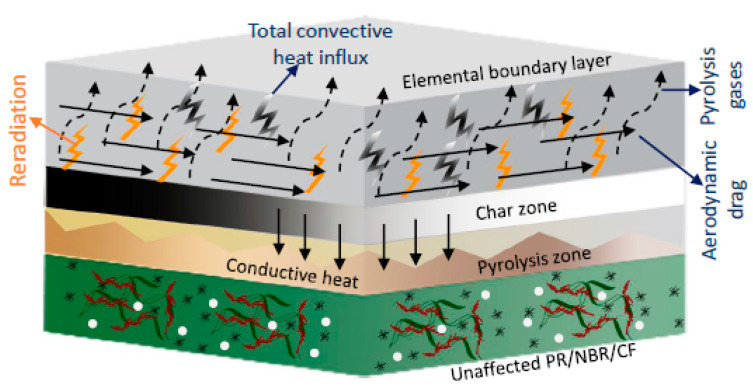
Ablation process/mechanism of PR/NBR/CF composite.

High-performance polymer ablative composites have a good ability to block, reflect, dissipate, and absorb surrounding bulk heat [19]. As the polymer ablative is subjected to a hyperthermal condition/environment, the temperature of the exposed surface rises to the thermal degradation temperature of the polymer, at which carbonaceous char is formed [20]. The efficiency of carbonization dictates the heat dissipation during ablation as it occurs as a result of blocking the penetration of heat from the pyrolyzed gas into virgin material by the charred surface [20]. Overall, the ablation process is complex and involves chemical reactions (endothermic and exothermic), heat transfer (conduction/convection) phase changes, and radiation and diffusion processes [20]. A schematic illustration of the ablation process/mechanisms is presented in Figure 7.

**Figure 8:**
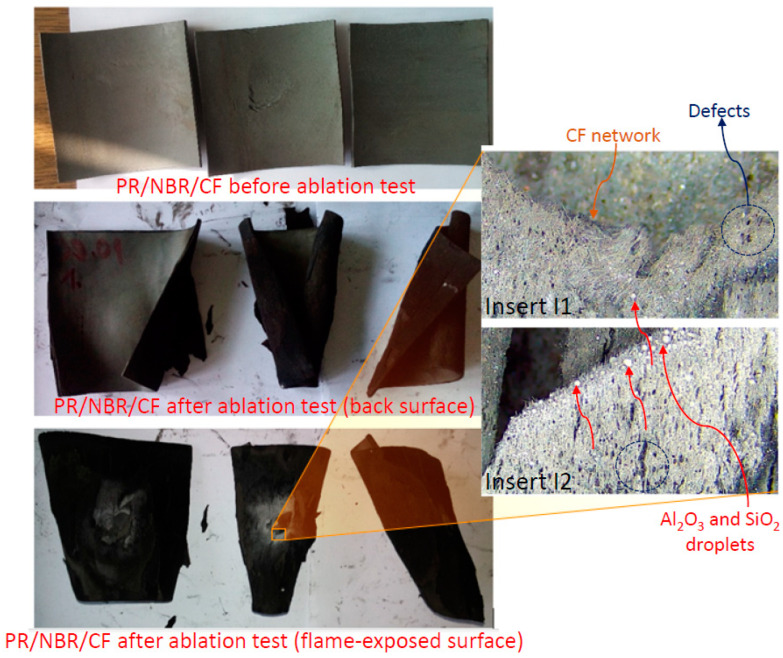
Sample of vulcanized PR/NBR/CF composite before and after ablation test (back surface and surface exposed to flame) and morphological analysis of PR/NBR/CF composite after ablation test with e-zoom of 30× (insert I1) and 100× (insert I2).

**Figure 9:**
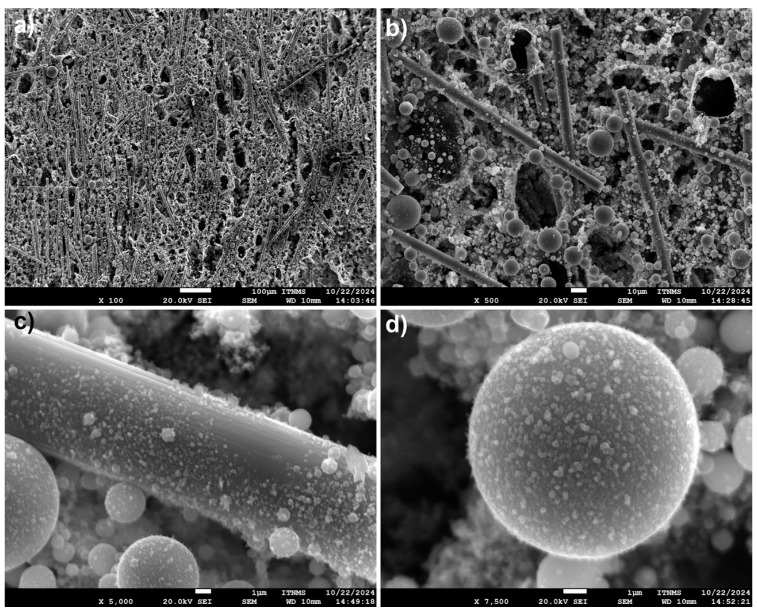
SEM micrographs of PR/NBR/CF composite after ablation test at different magnifications: (**a**) ×100, (**b**) ×500, (**c**) ×5000 and (**d**) ×7500.

The ablative performances can be considered according to the structure of the char layers formed in the ablation test [15]. The macroscopic appearance of the PR/NBR/CF composite before and after the ablation test, as well as the microscopic images of the carbonaceous char surface are shown in Figure 8, while the SEM micrographs are shown in Figure 9. It can be found that the designed PR/NBR/CF composite possesses good thermal/ablative performance since the formed char layer was not taken away during the ablation test, while the back surface of the composite stayed unaffected by the oxyacetylene flame for 15 s, which is twice as long as the operating time of a rocket motor. Apparently, both PR and NBR elastomer are components that promote carbonization in the developed thermal insulation according to the high char yield produced during the ablation test. Using fiber or mineral inorganic reinforcements in PR/NBR blends allows the achievement of high ablative performances as they absorb heat from the environment and cool the thermally exposed material. In addition, applied reinforcements provide high mechanical strength to the charred/carbonaceous material [17].

Morphological analysis of the surface of the char layer (Figure 8—inserts I1 and I2 and Figure 9) indicates that a negligible amount of defects, e.g., holes and cracks, are formed within the composite during the ablation test [15]. The high thermal conductivity of CFs promotes the thermal degradation of the NBR/PR matrix [15], resulting in enhanced char formation. CFs maintain a fibrous structure within the char layer after ablation, which can consolidate and connect carbonized NBR/PR matrix, preserving its structural integrity. Furthermore, powdered metal oxides such as fumed silica undergo many endothermic processes, such as melting and evaporation, thus acting as an additional heat sink (Equations (3) and (4) [21]). As a result of SiO_2_ melting and SiO_2_/CF interaction, a fibrous layer containing SiO_2_ droplets is formed on the ablated surface (Figure 8—inserts I1 and I2). SiO_2_ can slow down the oxygen diffusion and thermal conductivity, which prevent the oxidation of CF and other components of the NBR/PR matrix, indicating a high-performance ablative behavior with an erosion rate of 2.00 × 10^−4^ m·s^−1^. Conversely, Al_2_O_3_ as a filler with a high melting point of 2072 °C and excellent mechanical stability decreases linear and mass ablation rates when used in fiber-reinforced polymer composite materials [22]. The insulation index values at 80 °C and 180 °C are presented in Table 4 and demonstrate that the developed PR/NBR/CF composite possesses favorable ablative resistance.

SiO_2(S)_ → SiO_2(l)_ ΔH^Θ^_1713°C_ = 10 KJ mol^−1^ (3)

SiO_2(l)_ → SiO_2(g)_ ΔH^Θ^_2950°C_ = 761 KJ mol^−1^ (4)

### 3.4. Results of Firing of Solid Rocket Motor (SRM)

**Figure 10:**
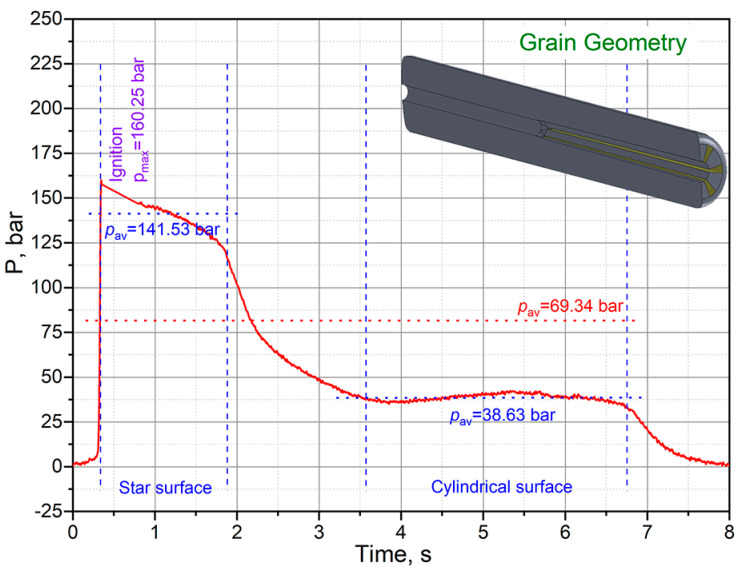
The pressure–time (P-t) diagram of AP/HTPB-based ACRP inhibited with developed PR/NBR/CF thermal insulation.

The propellant grain is a free-standing type, with a hybrid star–tube geometry configuration and an inhibited outer surface. Roughly half of the grain front is tube with a web/grain radius ratio of about 0.8, and the rear half has a classic star configuration. Such propellants’ geometry maximizes the burning surface area, providing a high thrust for initial launch of a missile in a two-phase rocket motor. The pressure–time (P-t) diagram provides insight into how the pressure develops in the combustion chamber over time during the rocket motor operation time. The P-t diagram of the experimental rocket motor consisting of AP/HTPB-based ACRP inhibited with developed PR/NBR/CF thermal insulation is shown in Figure 10. The ignition phase shows a rapid pressure rise (initial spike with the sharp peak at P-t diagram), after which, the pressure decreases until a pressure equilibrium is achieved. This slightly digressive phase occurs as the surface area remains consistent for the combined star–tube stage burning.

The gradual decrease in pressure can be observed as the shorter star web burns out and the burning surface area rapidly decreases. The pressure decrease continues until a sliver of star section burns out. In the second phase of the rocket motor operation, only the remaining cylindrical geometry burns, achieving an extended pressure plateau. Burning of the second phase provides enough thrust for a missile to sustain flight velocity. As the second phase burns out, the pressure decreases to 0. The total operational time of the experimental rocket motor was 7 s. The P-t diagram has the expected shape and values, so it can be concluded that there were no unpredicted increases in burning surface area during the whole rocket motor operational time. These conclusions are also supported by the rocket motor element inspection after disassembly. Such results indicate that PR/NBR/CF thermal insulation stayed mechanically stable and remained capable of withstanding the thermal and mechanical loads that occur during motor operation.

## 4. Conclusions

This work deals with a polymer matrix-based elastomer, novolac-type phenol–formaldehyde resin, elastomerized with nitrile rubber and reinforced with carbon fibers. The main function of the corresponding composite is the thermal protection of a steel rocket motor chamber during the combustion of the composite propellant. Given the above, mechanical and thermal properties of the prepared composite are tested and analyzed. Mechanical tests show that the obtained material has a rigid and brittle nature compared to similarly structured composites but good enough to withstand severe conditions during the exploitation of a rocket motor (high temperatures and pressure). In addition, the adhesion between the PR/NBR/CF-based composite inhibitor and ACRP is satisfactory, i.e., there is no de-bonding that could cause uncontrolled opening of the burning surface and thus, failure of the rocket motor. Thermal characteristics of the prepared composites, determined by ablation and firing tests, are suitable for the application of the corresponding composite as a TMP in a rocket motor. In brief, the low erosion rate, 2.00 × 10^−4^ m·s^−1^, high tensile strength, and elongation at break, 6.7 MPa and 419.92 MPa, respectively, indicate that the developed material can be applied as a thermal insulation/inhibitor of free-standing rocket propellant grains.

## Figures and Tables

**Table 1:** The PR/NBR/CF composite formulation.

Ingredients	PBW *	Wt.% **	mass, kg
NBR rubber, 28%	100	28.82	1.44
Phenolic resin	90	25.94	1.30
Carbon fiber	25	7.20	0.36
Carbon black	20	5.76	0.29
SiO_2_ (fumed silica)	45	12.97	0.65
Al_2_O_3_	50	14.41	0.72
Vulkacit H-30	8	2.31	0.12
Stearic acid	1	0.29	0.01
Vulkacit DM	1.5	1.44	0.07
Sulfur	1.5	0.43	0.02
ZnO	5.0	0.43	0.02
Total	347	100	5.0

* parts by weight; ** weight percentages.

**Table 2:** The values of stress at break (σ), max and elongation at break (ε_max_ and ε_br_), and tensile modulus (E) of developed PR/NBR/CF-based TPMs and comparison with literature data.

Tensile Test Results					
	σ, MPa	ε_max_, %	ε_br_, %	E, MPa	Reference
PR/NBR/CF	6.7 ± 0.5	418.3 ± 16.3	419.9 ± 15.9	7.1 ± 0.8	This work
PR_5PBW_/NBR	15.5	650.0	/	/	[11]
PR_10PBW_/NBR	19.5	575.0	/	/	[11]
PR_15PBW_/NBR	21.0	525.0	/	/	[11]
PR_20PBW_/NBR	18.2	495.0	/	/	[11]
NBR-Ph *	17.7	/	668.4	/	[14]

* Phenolic resin content—40 wt.%, carbon fabric (95% of carbon) layers—4.

**Table 3:** Bond strength between PR/NBR/CF and ACRP.

	T, °C	Load F, N	Strip Width, cm	Longitudal Load, N/cm
PR/NBR/CF	25	2.97	2.50	1.19

**Table 4:** The values of isolation indexes at 80 °C and 180 °C and erosion rate with literature comparison.

Sample	*I*_80_, s/m	*I*_180_, s/m	*E*, m/s	Reference
PR/NBR/CF	2262	4524	2.00 × 10^−4^	This work
PR_5PBW_/NBR *	/	/	2.25 × 10^−4^ **	[13]
PR_10PBW_/NBR *	/	/	2.00 × 10^−4^	[13]
PR_20PBW_/NBR *	/	/	1.17 × 10^−4^	[13]
PR_50PBW_/NBR *	/	/	1.00 × 10^−4^	[13]

* No filler added; ** Linear erosion rate.

## Data Availability

The original contributions presented in this study are included in the article. Further inquiries can be directed to the corresponding author.

## Abbreviations

The following abbreviations are used in this manuscript:

TPM	Thermal protective material
PR	Phenolic resin
NBR	Acrylonitrile butadiene rubber
CFs	Carbon fibers
PR/NBR/CF	NBR elastomerized PR composite, reinforced with chopped carbon fibers
PBW	Parts by weight
ACRP	Aluminized composite rocket propellant
DOA	Dioctyl adipate
TETA	Triethylenetetramine
IPDI	Isophorone diisocyanate
AP	Ammonium perchlorate oxidizer
